# Exploring the feasibility of combining transcutaneous electrical spinal cord stimulation and overground robotic exoskeleton for gait rehabilitation in patients with spinal cord injury

**DOI:** 10.3389/fneur.2025.1648616

**Published:** 2025-09-09

**Authors:** Marina Algaba-Vidoy, Soraya Pérez-Nombela, Álvaro Megía-García, Cristina Montero-Pardo, Carolina Redondo-Galán, Ana de los Reyes-Guzmán, Diego Serrano-Muñoz, Julio Gómez-Soriano, Antonio J. Del-Ama, Juan C. Moreno

**Affiliations:** ^1^BioRobotics Group, Centre for Automation and Robotics (CAR) CSIC-UPM, Arganda del Rey, Madrid, Spain; ^2^E.T.S. Ingenieros de Telecomunicación, Universidad Politécnica de Madrid, Madrid, Spain; ^3^Toledo Physiotherapy Research Group (GIFTO), Faculty of Physiotherapy and Nursing, Universidad de Castilla-La Mancha, Toledo, Spain; ^4^Toledo Physiotherapy Research Group (GIFTO), Instituto de Investigación Sanitaria de Castilla-La Mancha (IDISCAM), Toledo, Spain; ^5^Unit of Neurorehabilitation, Biomechanics and Sensorimotor Function (HNP-SESCAM), Associated Unit of R&D&I to the CSIC, Madrid, Spain; ^6^Neural Engineering Lab, Cajal Institute (CSIC), Madrid, Spain; ^7^Biomechanics and Technical Aids Unit, National Hospital for Paraplegics, Toledo, Spain; ^8^Bioengineering Systems and Technologies Research Group (BeST), Rey Juan Carlos University, Móstoles, Madrid, Spain

**Keywords:** exoskeleton, transcutaneous spinal cord stimulation, spinal cord injury, electromyography, locomotion rehabilitation, non-invasive neuromodulation

## Abstract

**Introduction:**

Spinal cord injury (SCI) is a traumatic condition that causes severe sensory and mobility impairments, including gait dysfunction. Combining exoskeleton-assisted gait training (EGT) with transcutaneous spinal cord stimulation (tSCS) may enhance motor recovery in SCI patients. This study explores the feasibility and immediate effects of combining these two neurorehabilitation strategies, without pursuing clinical benefits.

**Methods:**

We present a 3-of-1 case series of incomplete SCI patients (AIS C-D) who participated in two walking sessions: tSCS-assisted gait, then combined with the robotic exoskeleton Exo-H3. Each session included three phases of 3 min each: before, during and after tSCS. Surface electromyography (EMG) was recorded to analyze muscle activation and the level of effort generated using root mean square (RMS) and integrated EMG (iEMG). Functional outcomes were assessed with the Timed Up and Go (TUG) test, Visual Analog Scale (VAS) for discomfort and fatigue and distance covered.

**Results:**

Immediate effects varied among patients. Participant 1 showed increased muscle activation and effort without the Exo-H3 after tSCS, particularly in the Rectus Femoris (ReFe) muscle, along with improved TUG performance and walking speed. However, during the combined tSCS-Exo session, muscle activation did not decrease, but effort was significantly reduced, masking the tSCS effects seen without the exoskeleton. Walking speed and TUG also worsened. Participant 2 exhibited reduced RMS and iEMG in both conditions, especially in the ReFe, with no notable improvement in TUG score or distance covered. In contrast, Participant 3 appeared to benefit from both sessions, showing increased activation and exertion in the tibialis anterior and upper leg (biceps femoris and ReFe). TUG did not improve in the non-exoskeleton session after tSCS but showed significant improvement when combined with Exo-H3. None of the participants reported abnormal discomfort or fatigue beyond the levels typically associated with exoskeleton use.

**Discussion:**

The combined use of tSCS and EGT appears technically feasible and safe, whereas the responses were highly individualized. Knee extensors muscles showed the greatest responsiveness to tSCS during gait. Synergistic effects may depend on the user’s proficiency with the exoskeleton. Further analysis and larger studies are needed to better identify SCI who may benefit the most.

## Introduction

Spinal cord injury (SCI) is a devastating neurological condition that involves damage or dysfunction of the spinal cord, resulting in partial or complete loss of sensation and motor control below the level of the injury. The incidence of SCI varies globally, with an estimated 250,000 to 500,000 new cases occurring worldwide each year. The economic cost associated with SCI in the U.S. amounts to $10 billion annually (1). SCI has wide-ranging consequences, with the loss of walking ability being one of the most significant.

Although wearable powered exoskeletons have emerged as promising tools for gait assistance and rehabilitation, especially in individuals with SCI (2, 3), their therapeutic effectiveness remains uncertain. In (3), they stated that there is currently no evidence that robot-assisted gait training improves walking function more than other locomotion training strategies. Likewise, the evidence reviewed in (2), primarily based on observational studies, suggests a need for further research to strengthen the conclusions. Despite the ability of exoskeletons to provide external support and facilitate controlled, repetitive movements (4), their potential for promoting meaningful motor recovery is still under debate (5).

The focus on restoring and enhancing gait control in individuals with neurological disabilities has indeed driven researchers to explore advanced methods that activate spinal circuits involved in locomotion (6, 7). Transcutaneous electrical spinal cord stimulation (tSCS) is a non-invasive approach aimed at modulating the central nervous system, specifically at the spinal cord level, to influence neural excitability (8, 9). This technique involves the application of electrical currents through surface electrodes usually placed over the T11-T12 vertebrae (10). While early studies have suggested the potential of tSCS to enhance voluntary motor response, trunk stability and functional outcomes in individuals with SCI (9, 11), the limited samples sizes and preliminary nature of these reports highlight the need for further validation. Nonetheless, more recent evidence from a randomized controlled trial has demonstrated promising results regarding its therapeutic efficacy (12), emphasizing the importance of continuing to explore its potential.

The existing evidence underscores the need to explore complementary strategies, such as the combination of exoskeleton gait training (EGT) with tSCS, which offers an exciting therapeutic approach. This combination is believed to enhance the potential for motor recovery, as sensory input during movement can reinforce spinal circuits that control gait patterns (13). Furthermore, robotic technologies, such as exoskeletons, can serve as an effective stimulus for rehabilitation, potentially increasing patient engagement and adherence to treatment compared to conventional rehabilitation methods. Although the current evidence is limited, preliminary results suggest promising outcomes from combining EGT and tSCS treatment in individuals with SCI. The study with the largest sample size to date, by (14), included 19 individuals with complete SCI who completed a 2-week program of combined EGT with tSCS, resulting in a significant increase in foot loading forces. In a separate case study (13), combined tSCS with pharmacological treatment and EGT, which enhanced the level of effort and improved coordination patterns of the lower limb muscles, producing a continuous stepping motion in the exoskeleton. Lastly, only one study (15) provided electromyography (EMG) data, observing that combining tSCS and EGT in three individuals with SCI resulted in individualized responses, but generally increased knee extensor activity, and two out of the three participants were able to initiate more steps without additional assistance from the exoskeleton. Additionally, a key advantage of robotic exoskeletons is their potential to be combined with other therapeutic interventions, such as tSCS, thereby enhancing the overall rehabilitation process.

While the combination of tSCS and EGT has been explored in previous studies with promising results, important methodological and translational questions remain unresolved – particularly in real-world clinical contexts and in relation to immediate neuromuscular effects. This work aims to modestly contribute to this ongoing discussion by providing additional clinical observations from a small, heterogeneous group of individuals with SCI. The motivation for this study lies in the scarcity of scientific literature and the heterogeneity observed in the response of patients with SCI to rehabilitation therapies. The results of the application of tSCS and EGT vary significantly between patients, highlighting the need for individualized rehabilitation strategies. Thus, the objective of this study is to help illustrate potential effects and practical considerations of this hybrid therapy in real-world clinical settings. More specifically, it seeks to: (i) describe short-term observations related to the potential and limitations of the combined application of tSCS and EGT; (ii) complement with the analysis of EMG data to gain deeper insight into the immediate physiological effects of the therapy; and (iii) share relevant clinical and technical insights to inform future studies and contribute to the gradual understanding of how tSCS interacts with EGT in individuals with SCI. It is important to note that this study is not intended to produce a significant clinical impact in a single session. Instead, it aims to assess the feasibility and clinical viability of applying a hybrid approach in a real-world rehabilitation context with patients. The goal is to explore its potential therapeutic value as a starting point for future research with larger and more diverse samples.

## Materials and methods

### Participants

This article presents a series of single cases (3-of-1 study) of three volunteers with incomplete motor SCI (AIS C-D), with an evolution of at least 2 months, neurological level between C4 and T11, aged over 18 years and with the ability to understand and follow instructions. Some of the exclusion criteria were intolerance to electrostimulation, peripheral neurological injury in the lower limbs, history of epilepsy, vertebral arthrodesis in the stimulation area, severe spasticity (modified Ashworth and/or Penn scores >3), weight over 100 kg and height over 180 cm. The clinical data of the participants are shown in Table 1. All voluntary participants were recruited from the National Hospital for Paraplegics in Toledo and signed informed consent before participating in the study, which was approved by the Ethics Committee for Clinical Research of the Área Sanitaria de Toledo (Approval number: 918, date: 09/11/2022).

**Table 1:** Demographic data.

Participant	Age	Sex	BMI (kg/m^2^)	Months post-injury	Level of injury	AIS
1	60	Male	26.53	6	T6	C
2	69	Male	21.97	7	C4	D
3	66	Male	24.49	2	T11	C

BMI, Body Mass Index; AIS, ASIA Impairment Scale.

The functional characteristics of the three patients have been described using several standardized measures. The Lower Extremity Motor Score (LEMS) subscale (0–50) was used to assess lower limb motor strength and to identify the most affected side of the patient (16). Hypertonia was assessed with the Modified Ashworth Scale (17), spasms were quantified using the Penn Spasm Frequency Scale (18) and walking speed was evaluated using the 10-meter walk test (10MWT) (19). The level of independence in daily living activities was assessed using the Spinal Cord Independence Measure III (SCIM III) (20). This scale comprises 19 items and higher scores indicate a greater degree of independence. Additionally, the Walking Index for Spinal Cord Injury II scale (WISCI-II) was used to score patients on a scale from 0 to 20, considering the technical aids, orthoses and assistance required for walking 10 m (21). All these data are presented in Table 2.

**Table 2:** Clinical data.

Participant	LEMS		Most affected side	Ashworth		Penn	10MWT	WISCI-II	SCIM-III
	Left	Right		Left	Right				
1	21	13	Right	2	2	2	22	12	46
2	17	12	Right	0	0	0	47	9	56
3	16	18	Left	0	0	0	13.6	13	70

LEMS, Lower Extremity Motor Score; 10MWT, 10-meter walking test; WISCI-II, Walking index for spinal cord injury; SCIM-III, Spinal cord independence measure.

### Robotic system

The robotic exoskeleton utilized in this study was the Exo-H3 (Technaid S. L., Arganda del Rey, Spain). The Exo-H3 is equipped with six actuators composed of DC motors and harmonic gears located at the hip, knee and ankle joints on both sides. It offers multiple control modes: Passive Mode, allowing free user movement without assistance, suitable for assessing natural mobility; Compliance Mode, providing limited assistance with controlled resistance to assess muscle strength; Assistive Mode, actively aiding in walking and specific exercises by significantly reducing user effort; and Resistance Mode, which applies opposing force to enhance muscle strength and endurance, primarily for advanced rehabilitation. It can interface with external devices through CAN bus, Bluetooth or WiFi, transmitting exoskeleton parameters—including joint angles, interaction forces at the foot, leg and thigh segments and foot-ground contact—at a frequency of 100 Hz (22).

### Transcutaneous spinal cord stimulation

**Figure 1:**
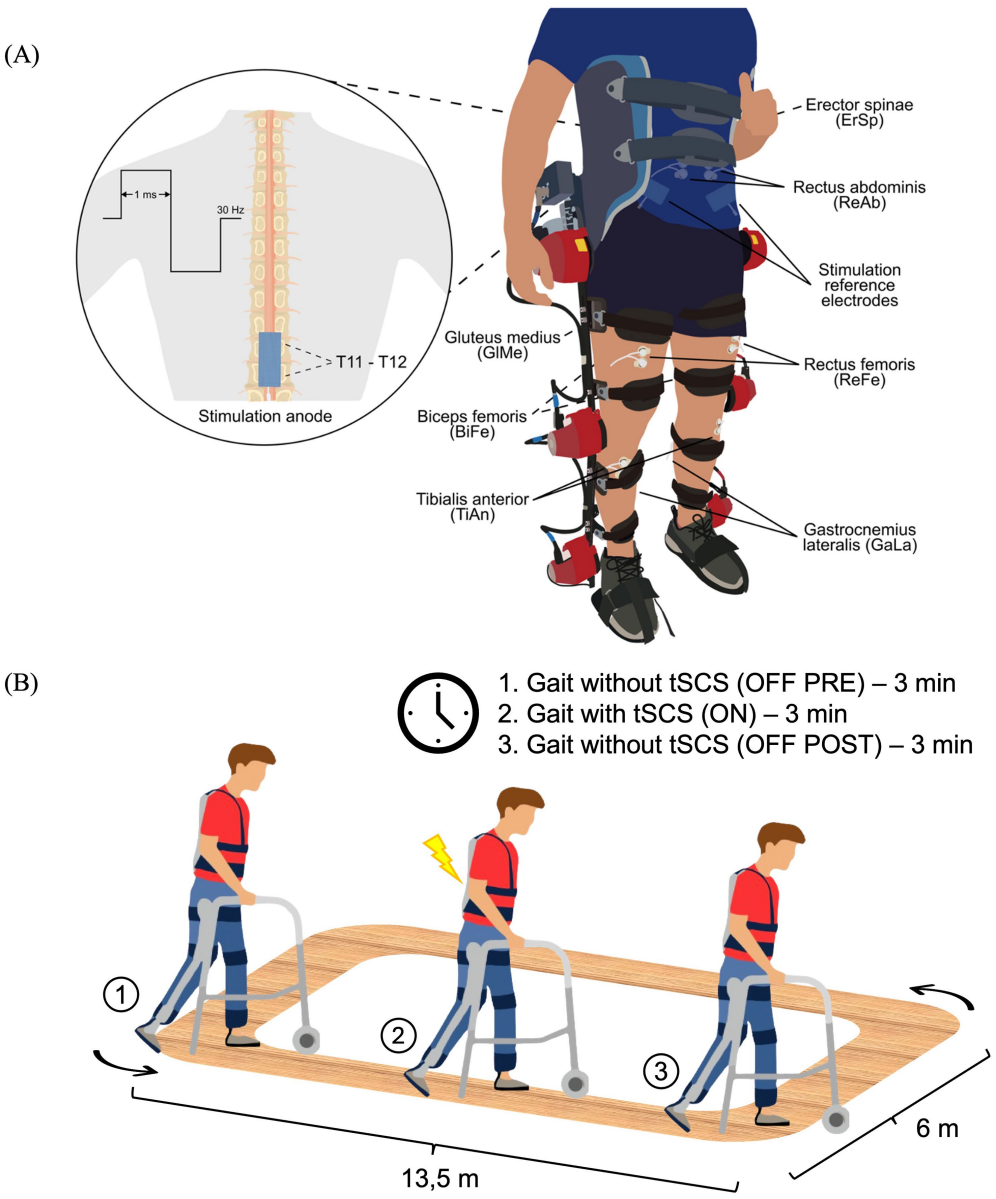
Schematic representation of the experimental setup **(A)** and experimental protocol **(B)**. **(A)** Bipolar EMG electrodes were placed over the following muscles: Erector Spinae (ErSp), Rectus Abdominis (ReAb), Gluteus Medius (GlMe), Rectus Femoris (ReFe), Biceps Femoris (BiFe), Tibialis Anterior (TiAn) and Gastrocnemius Lateralis (GaLa). For the application of tSCS, reference electrodes were placed over the iliac crests and the stimulation anode was positioned at the T11-T12 spinal level, delivering a waveform at 30 Hz with a 1 ms pulse width. **(B)** The protocol consisted of two randomized sessions (with and without the Exo-H3), each divided into three phases: 3 min of gait without stimulation (OFF-PRE), 3 min of gait with tSCS (ON) and 3 min of gait after stimulation (OFF-POST). Exo-H3, exoskeleton; tSCS, transcutaneous spinal cord stimulation.

tSCS was applied to all subjects using the “Myomed 932” stimulator by Enraf-Nonius (Rotterdam, the Netherlands) in an open-loop system (without communication with the Exo-H3). A symmetric biphasic rectangular current was selected, with a frequency of 30 Hz and a pulse width of 1 ms. A single stimulation channel was used, with the anode placed on the interspinous line at T11-T12 and two reference electrodes positioned on the iliac crests. This procedure is systematically represented in Figure 1A. Self-adhesive pre-gelled flexible carbon electrodes (9×5 cm, ValuTrode, Axelgaard Manufacturing Co., USA) were used. The stimulation intensity was individually adjusted in each session, starting from 0 mA and gradually increasing up to the maximum level that was tolerable for the patients without causing discomfort. This approach, while limiting standardization across participants, was chosen to ensure safety and clinical applicability, as the optimal stimulation intensity for tSCS remains undetermined. At this threshold, patients commonly reported experiencing paresthesia in the lower limbs. The parameters were based on those used in this previous study (23) and stimulation location followed the protocol described by (24), as the anode could not be placed at the umbilical level due to the presence of EMG electrodes for the ReAb.

### Study protocol

Two independent study sessions were conducted on separate days in a randomized order. One session consisted of walking with their usual assistive devices at their self-selected speed for a total time of 9 min. The other session also involved walking for 9 min with the Exo-H3 in Assistive Mode (100% of symmetrical assistance) at the minimum gait cycle speed set to 4.5 s (speed level 1) to provide high assistance during gait. Both sessions were divided into three blocks of 3 min each: OFF-PRE represents the 3 min period before tSCS, ON corresponds to the intermediate period of 3 min during which tSCS was applied and OFF-POST refers to the 3 min following the application of tSCS. The participants could rest as needed between each trial to avoid fatigue. The walking sessions took place on a flat and rectangular terrain as depicted in Figure 1B. Prior to beginning the study protocol, participants underwent separate familiarization sessions with both the tSCS and the Exo-H3. The tSCS session was specifically focused on familiarizing participants with the stimulation device. The duration of the Exo-H3 training varied by patient, with individuals considered familiarized once they were able to walk continuously for 3 min using the exoskeleton.

Before starting each session, with the patient lying on a treatment table, stimulation electrodes were positioned for spinal stimulation and the current intensity was set. In this same position, the skin was prepared and surface EMG electrodes were placed to record muscle activity.

## Assessment

### EMG recordings

Prior to electrode placement, the skin was carefully prepared by cleansing with alcohol and shaving the areas designated for electrode application to ensure optimal signal quality. The reference electrode was moistened to enhance conductivity and positioned on the ankle. Muscle activity was recorded with Ag/AgCl bipolar electrodes. They were placed in seven muscles bilaterally following SENIAM recommendations (25): erector spinae (ErSp), rectus abdominis (ReAb), gluteus medius (GlMe), ReFe, biceps femoris (BiFe), gastrocnemius lateralis (GaLa) and tibialis anterior (TiAn), as illustrated in Figure 1A. However, due to high levels of electrical noise, some muscles were finally excluded from the analysis. All electrodes were connected to the Quattrocento amplifier from OT Bioelettronica (Torino, Italy) with an acquisition frequency of 2,048 Hz.

### Functional assessment

To determine the immediate effects of the tested conditions on the patient’s balance and walking, the Timed Up and Go (TUG) test (19) was administered before and after each session. In addition, discomfort and fatigue were recorded using the Visual Analog Scale (VAS) after each session and the distance covered in meters under each of the two conditions.

### EMG processing

EMG signals required meticulous processing due to the high level of noise and interference introduced by the experimental setup. Data were processed in MATLAB R2023b (The MathWorks, Natick, MA). Firstly, EMG signals were band-pass filtered (2nd order Butterworth 20–500 Hz). Power line interference was removed with a notch filter at 50 Hz. Additionally, to minimize artifacts from the tSCS, zeros were inserted at the harmonics of the stimulation frequency using a custom filter designed with the pole-zero placement method (26). Signals were then smoothed with a median filter to mitigate remaining artifacts within the frequency of activation, whether caused by interferences with the Exo-H3 or by cable movement. Finally, envelopes were computed with a low-pass filter at 1 Hz (2nd order Butterworth). The cut-off frequency was adjusted to the patients’ walking speed, which was notably slow, as higher frequencies did not accurately represent their walking patterns (27).

The EMG signals and the muscles to be used in each case were selected through visual inspection by expert researchers in the field, discarding signals where the walking pattern could not be visually identified. Gait cycles were segmented using an algorithm based on Dynamic Time Warping applied to EMG signals (28), which was originally developed for gait segmentation using inertial sensor data (29). Once gait cycles were extracted, the corresponding EMG envelopes were normalized with respect to the mean of the peaks from all gait cycles and they were visually inspected to select the most representative cycles. At least 10 cycles were ensured.

EMG Root Mean Square (RMS) and integrated EMG (iEMG) were calculated for each cycle. For each session separately (without Exo-H3 and with Exo-H3), OFF-PRE and OFF-POST values were compared within each subject using a Wilcoxon rank-sum test to assess significant differences due to the immediate effect of the application of tSCS and the Exo-H3. The two sessions were not analyzed together, as they were performed on different days and under different circumstances, making such comparison questionable. A significance of *p*-value < 0.05 was considered. The statistical analysis was also performed in MATLAB R2023b (The MathWorks, Natick, MA).

## Results

In all participants, the ErSp and ReAb muscles were excluded from the analysis due to consistent protocol-related difficulties. Specifically, collected data were deemed unusable, as recordings contained no discernible signal (i.e., surface electrodes experienced persistent detachment, likely caused by perspiration or friction with the Exo-H3; or failed to register due to variations in subcutaneous fat composition) or were dominated by electrical noise, primarily during current application.

**Figure 2:**
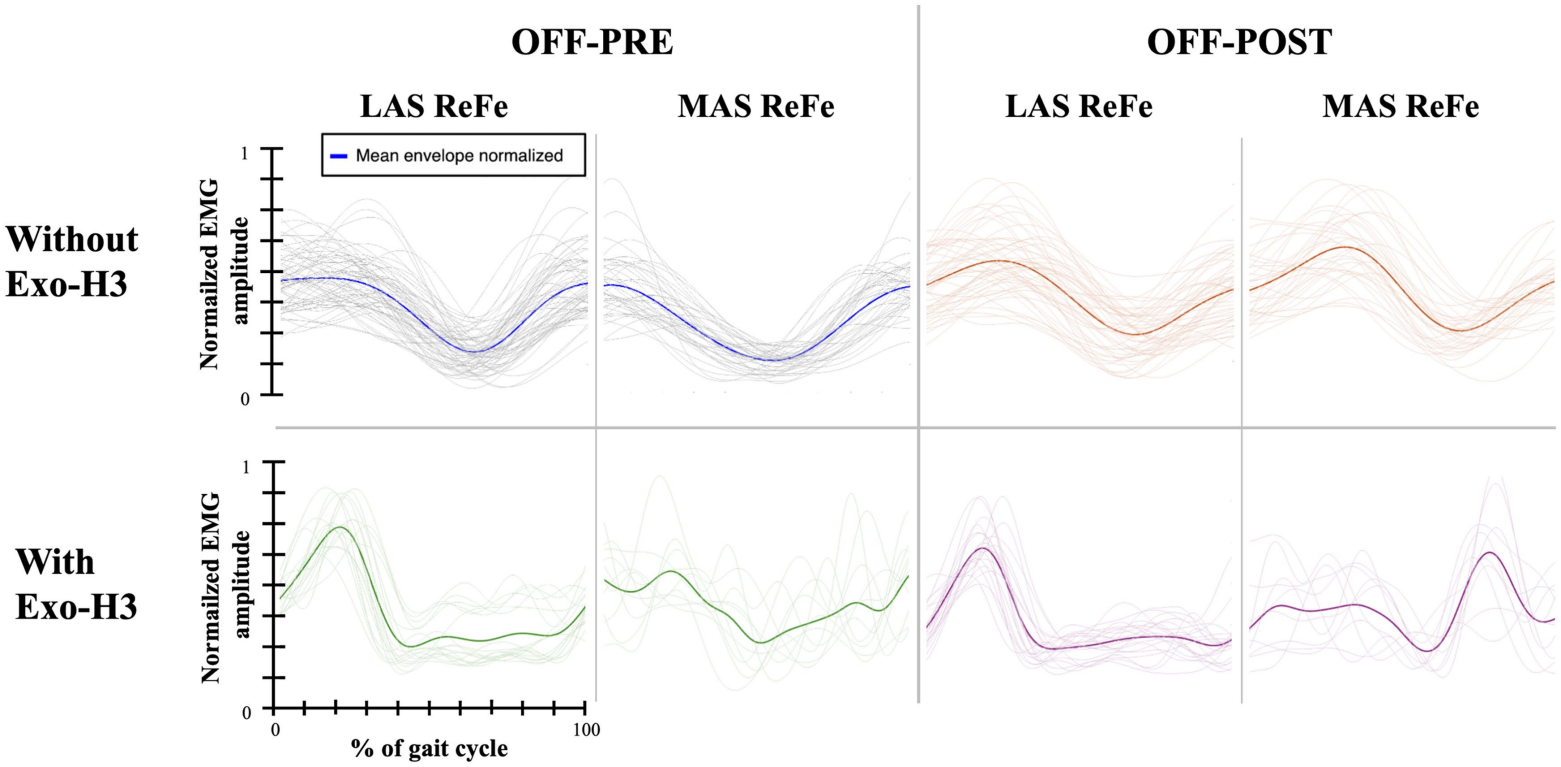
EMG envelopes and mean envelope (highlighted with a thicker line) across the gait cycle for Participant 1. Data are shown for the ReFe muscle on both the less affected side (LAS) and most affected side (MAS) under four conditions: OFF-PRE without Exo-H3 (blue), OFF-POST without Exo-H3 (orange), OFF-PRE with Exo-H3 (green) and OFF-POST with Exo-H3 (purple). The X- and Y-axes are consistent across all plots. EMG, electromyography; Exo-H3, exoskeleton device; ReFe, rectus femoris; tSCS, transcutaneous spinal cord stimulation; OFF-PRE, walking trial before the application of tSCS; OFF-POST, walking trial after the application of tSCS.

**Figure 3:**
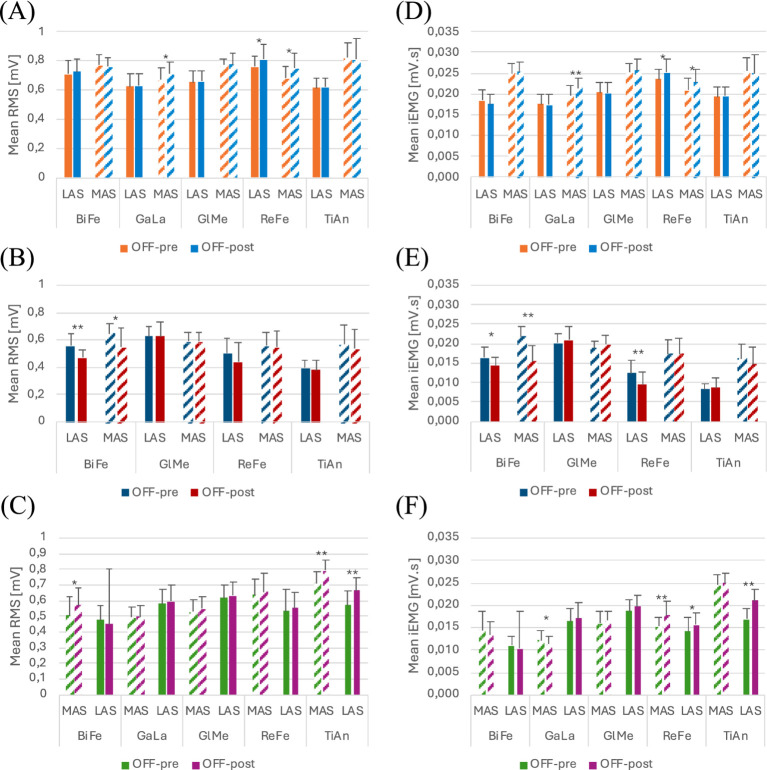
Plots of the mean and standard deviation of RMS (left) of **(A)** Participant 1, **(B)** Participant 2 and **(C)** Participant 3 and iEMG (right) of **(D)** Participant 1, **(E)** Participant 2 and **(F)** Participant 3 for both OFF-PRE and OFF-POST conditions in the session without Exo-H3. * *p* < 0.05; ** *p* < 0.001. The position of LAS and MAS is related to whether the less or most affected side correspond to the left or right side of each participant. RMS, Root Mean Square; iEMG, integrated EMG; tSCS, transcutaneous spinal cord stimulation; OFF-PRE, walking trial before the application of tSCS; OFF-POST, walking trial after the application of tSCS; Exo-H3, exoskeleton device; LAS, less affected side; MAS, most affected side.

**Figure 4:**
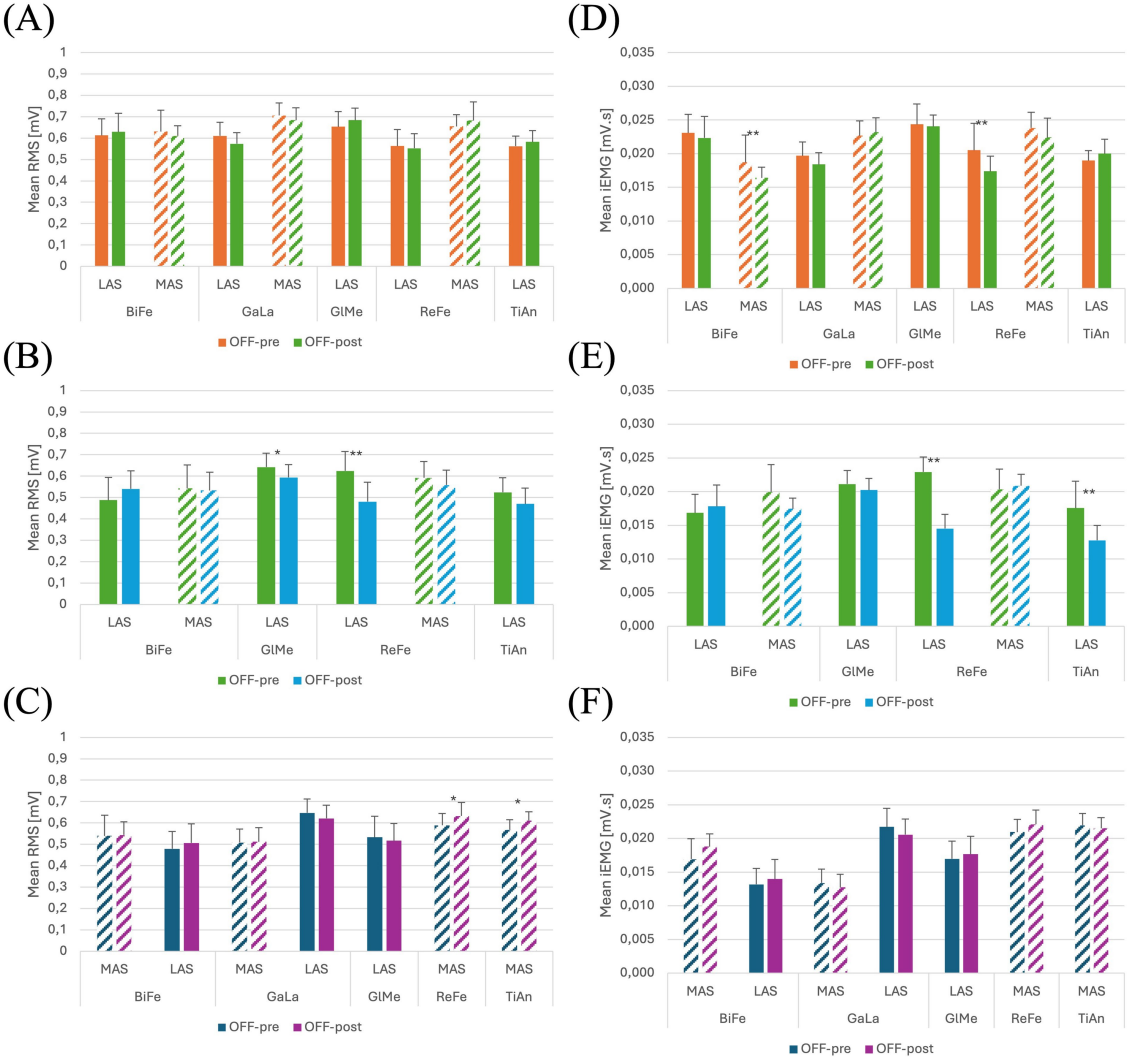
Plots of the mean and standard deviation of RMS (left) of **(A)** Participant 1, **(B)** Participant 2 and **(C)** Participant 3 and iEMG (right) of **(D)** Participant 1, **(E)** Participant 2 and **(F)** Participant 3 for both OFF-PRE and OFF-POST conditions in the session with Exo-H3. * *p* < 0.05; ** *p* < 0.001. The position of LAS and MAS is related to whether the less or most affected side correspond to the left or right side of each participant. RMS, Root Mean Square; iEMG, integrated EMG; tSCS, transcutaneous spinal cord stimulation; OFF-PRE, walking trial before the application of tSCS; OFF-POST, walking trial after the application of tSCS; Exo-H3, exoskeleton device; LAS, less affected side; MAS, most affected side.

Functional assessment data (VAS discomfort and fatigue, TUG and covered distance) are presented in Table 3. Walking speeds for each trial are listed in Table 4. Table 5 presents a summary of the statistically significant results for each subject regarding the RMS and iEMG values in the OFF-PRE compared to the OFF-POST condition for both sessions, with changes expressed as percentages of change. An example figure illustrating the EMG envelopes for Participant 1’s ReFe muscle, including both the most affected and less affected sides across all conditions (OFF-PRE and OFF-POST) and both sessions (with and without Exo-H3), is included for detailed comparison (Figure 2). Figures 3, 4 display comparative graphs of the mean values and the standard deviation for RMS and iEMG across both conditions (without Exo-H3 and with Exo-H3, respectively) for each subject.

**Table 3:** Functional assessment.

Session		Participant 1	Participant 2	Participant 3
Without Exo-H3	Discomfort VAS	0	0.6	4.9
	Fatigue VAS	0.5	3.4	0
	TUG-PRE (s)	24.5	68	32
	TUG-POST (s)	23.2	65	32
	Distance (m)	232	85.5	181.7
	Order	2°	2°	1°
With Exo-H3	Discomfort VAS	0	0.5	3
	Fatigue VAS	2.6	4.6	4.4
	TUG-PRE (s)	23.16	72	28
	TUG-POST (s)	25.4	69	20
	Distance (m)	98.25	82.75	105
	Order	1°	1°	2°

Exo-H3, exoskeleton device; VAS, Visual Analog Scale; TUG, Timed up and go; PRE, before the session begins; POST, after the session ends.

Order indicates the randomized order in which the sessions with and without Exo-H3 were conducted for each participant.

**Table 4:** Walking speeds (in meters per seconds, m/s) for each trial.

Walking speed (m/s)	Without Exo-H3				With Exo-H3			
	OFF-PRE	ON	OFF-POST	Mean walking speed	OFF-PRE	ON	OFF-POST	Mean walking speed
Participant 1	0.415	0.443	0.398	0.419	0.128	0.089	0.174	0.130
Participant 2	0.114	0.153	0.164	0.144	0.159	0.109	0.145	0.138
Participant 3	0.273	0.266	0.302	0.208	0.176	0.171	0.165	0.171

Exo-H3, exoskeleton device; tSCS, transcutaneous spinal cord stimulation; OFF-PRE, walking trial before the application of tSCS; ON, walking trial during the application of tSCS; OFF-POST, walking trial after the application of tSCS.

**Table 5:**
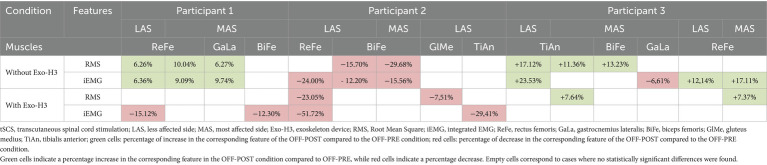
Results of the statistical comparison between the OFF-PRE (walking trial before the application of tSCS) and OFF-POST (walking trial after the application of tSCS) conditions, expressed as percentage increases or decreases.

### Participant 1

Participant 1 was a 60-year-old man with a BMI of 26.53 kg/m^2^ and a SCI at T6 level, with 6 months from the injury and AIS C (Table 1). As shown in Table 2, the right lower limb was the most affected (LEMS = 13), modified Ashworth of 2 in both limbs, Penn of 2, the score of the WISCI-II was 12 points (ambulates with two crutches, with braces and no physical assistance, 10 meters) and SCIM-III was 46 points. 22 s were taken to walk 10 meters.

#### Without Exo-H3

As it can be seen in Figure 3A, RMS increased in both the less affected and most affected ReFe, as well as in the most affected GaLa, in the OFF-POST compared to the OFF-PRE condition while walking without robotic assistance. Specifically, the less affected ReFe and most affected ReFe experienced an increase of 6.26 and 10.04%, respectively, while the most affected GaLa showed a rise of 6.27%. iEMG (Figure 3D) was also augmented in the less affected ReFe (6.36%), most affected ReFe (9.09%) and most affected GaLa (9.74%). Likewise, walking speed was greater during the ON phase and the time taken to perform TUG test after the application of tSCS decreased by 1.3 s. For this participant, discomfort VAS indicated a score of 0 and fatigue VAS was 0.5. The distance covered in this session was 232 meters.

#### With Exo-H3

For this session, most affected GlMe and TiAn were removed from the analysis, due to their suboptimal quality, which was confounding the results. In contrast, the iEMG values in the OFF-POST condition were lower than those in the OFF-PRE in the less affected ReFe (decreased by 15.12%) and most affected BiFe (reduced by 12.30%). This can be observed in Figure 4D. Walking speed was almost the same in the OFF-PRE (0,128 m/s) and the OFF-POST (0,174 m/s) phases. However, mean walking speed was substantially worsened when using the Exo-H3 (0.13 m/s) compared to not using it (0.419 m/s), with a difference of 0.288 m/s. A similar trend was observed in the timing for the TUG test, with an increase from 23.16 s before stimulation to 25.4 s after (a difference of 2.24 s). However, these results were not reflected in the RMS, as no significant changes were found (Figure 4A). In comparison to the session without Exo-H3, discomfort VAS indicated a score of 0 before the activity and increased 0.5 cm after the task. The fatigue VAS showed a similar trend, rising from 0 to 2.6 cm. The distance covered with the robotic assistance diminished to 98.25 meters.

### Participant 2

Participant 2 was a 69-year-old man with a BMI of 21.97 kg/m^2^ and a SCI at C4 level, with 7 months from the injury and AIS D (Table 1). As shown in Table 2, the right lower limb was the most affected (LEMS = 12), without hypertonia nor spasms (modified Ashworth and Penn scores of 0) and a WISCII-II score of 9 points (ambulates with walker, with braces and no physical assistance). The SCIM-III score was 56 points and he took 47 s to walk 10 meters. For patient two, the GaLa was excluded from the analysis due to insufficient signal quality.

#### Without Exo-H3

As shown in Figure 3B, RMS was reduced in the OFF-POST condition without Exo-H3 for the less affected (decreased by 15.70%) and most affected BiFe muscle (decreased by 29.68%). iEMG values for the less affected ReFe (decreased by 24.00%), less affected BiFe (decreased by 12.20%) and most affected BiFe (decreased by 15.56%) also exhibited a decline (Figure 3E). Walking speed remained unchanged after the stimulation (difference of 0.05 m/s, Table 4), but TUG performance was improved by 3 s following the tSCS. The patient reported discomfort and fatigue VAS scores of 0.6 and 3.4 cm, respectively, while covering a distance of 85.5 meters during this session.

#### With Exo-H3

To perform this analysis, most affected GlMe and TiAn muscles were excluded. In Figure 4B, a decrease can be observed in RMS values for the less affected ReFe (decreased by 23.05%) and the less affected GlMe (decreased by 7.51%) and in iEMG values for the less affected ReFe (decreased by 51.72%) and less affected TiAn (decreased by 29.41%) in the OFF-POST with Exo-H3 condition (Figure 4E). However, mean walking speeds remain almost the same between the Exo-H3 (0.138 m/s) and the non-Exo-H3 condition (0.144 m/s). Walking speed was maintained after the ON phase (OFF-PRE = 0,159 m/s; OFF-POST = 0,145 m/s), but TUG was reduced in 3 s. VAS indicated a discomfort level of 0.5 cm and fatigue of 4.6 cm.

### Participant 3

The last participant (male, 66 years old and BMI of 24.49 kg/m^2^) suffered from a spinal cord lesion at C4 level (AIS D) 2 months before the assessment. As shown in Table 2, he had the left lower limb more affected (LEMS = 16) and a WISCI-II of 13 (ambulates with walker, no braces and no physical assistance, 10 meters). It took 13.6 s to walk 10 meters and had a SCIM-III score of 70 points.

#### Without Exo-H3

For participant 3, an increase in the RMS outcome can be appreciated in Figure 3C. On the less affected side, this increase was 17.12% for the TiAn, while on the most affected side, it was 11.36% for the TiAn and 13.23% for the BiFe. However, for the iEMG variable, as shown in Figure 3F, an increase of 23.53% was noted in the TiAn and 12.14% in the ReFe on the less affected side. In contrast, on the most affected side, the increase was significant only in the ReFe at 17.11%, while a decrease of 6.61% was observed in the GaLa. TUG was unaltered in the OFF-POST phase, while walking speed slightly increased from 0.273 m/s to 0.302 m/s. The patient reported a discomfort score of 4.9 cm based on the VAS, without indication of perceived fatigue. This participant walked a distance of 181.7 meters during this session.

#### With Exo-H3

In this case, the less affected ReFe and TiAn, along with the most affected GlMe, were removed to prevent their noise and poor signal quality from obscuring the other results.

In the session with Exo-H3, only RMS values varied significantly in the less affected TiAn, increasing a 7.64% and less affected ReFe with a 7.37% (Figures 4C,F). Walking speed did not change compared with Exo-H3 OFF-PRE (0.176 m/s) and with Exo-H3 OFF-POST (0.165 m/s).

Yet, TUG was clearly improved in 8 s after the ON phase and a great improvement existed between the TUG performance without Exo-H3 and with Exo-H3. Indeed, mean walking speed also went from 0.2081 m/s without Exo-H3 to 0.1705 m/s with Exo-H3.

Notably, discomfort was lower compared to the session without robotic assistance (discomfort VAS = 3 cm), but VAS fatigue was increased to 4.4 cm. The distance was reduced to 105 meters.

## Discussion

Overall, the results show the heterogeneity that exists in the responses of each participant with SCI, highlighting that rehabilitation strategies should be tailored individually. Our findings support the hypothesis that studying and implementing the most appropriate rehabilitation approach for each patient is essential for an effective treatment. In particular, acute neuromuscular responses (i.e., EMG activity patterns) may serve as early indicators of how individuals respond to the intervention, offering a potential basis for patient stratification or for personalization of stimulation and training parameters. It must be acknowledged that this investigation was limited to a single session, therefore a significant clinical impact or improvement was not expected. Having said that, the primary objective was to evaluate the clinical feasibility of integrating these two potential rehabilitation strategies, assessing the practicality and plausibility of applying them in realistic clinical settings. By exploring the application in a real-world clinical environment, the study offers a starting point and contributes practical insights regarding how such hybrid intervention could be further implemented and adapted to patient-specific needs.

Participant 1 showed a high level of proficiency in walking independently without the Exo-H3, with improved walking speed and TUG score. tSCS seemed to enhance activation in the ReFe and GaLa muscles, potentially benefiting gait performance. This aligns with the increase in iEMG values observed in the ReFe and gastrocnemius medialis in another study (13). Furthermore, this supports previous findings demonstrating that the knee extensor muscles are among the most influenced by tSCS (15, 23). As previously stated, both RMS and iEMG exhibited an increase, potentially indicating a greater level of muscle activation and an enhancement in the effort the subject could generate while walking, respectively. This suggests that, for this patient, tSCS may have modulated the excitability of the spinal neural networks to a higher state, resulting in higher EMG activity patterns in certain lower extremity muscles.

Conversely, iEMG values after the application of tSCS in the session with Exo-H3 were reduced in the less affected ReFe and the most affected BiFe, while no change was observed in RMS.

We have noticed that the patient seemed to be relying on the exoskeleton for walking, instead of actively engaging in the walking process. This is evident from the lack of change in RMS (no increase in activation parameters) and the decrease in iEMG, indicating that the effort the patient exerts while walking is reduced, resulting in less involvement in the locomotor task. In addition, both walking speed and distance covered were significantly lower with the Exo-H3 compared to the session without Exo-H3. However, the effect is not counterproductive: the Exo-H3 is simply not contributing to the improvement of muscle activation patterns during walking. This can be asserted as the patient did not express any discomfort or significant fatigue while using the Exo-H3 according to the VAS scale. Consequently, the absence of improvement could be related to insufficient familiarization or motivation to use the assistive device. It may also suggest that the Exo-H3 did not generate a real assistive effect in which the patient’s gait is improved through more active participation in the walking task. Instead, in patients with a slightly more functional baseline gait, the device might lead them to become more passive, letting themselves be carried by the exoskeleton without actively engaging the movement. This reduced level of involvement may be masking the effects of tSCS that were observed during the session without Exo-H3.

Participant 2 exhibited significant challenges with independent ambulation, as evidenced by the poor performance in TUG test and the short distances covered during both sessions. The application of tSCS during walking without exoskeleton did not produce a significant enhancement in muscle activation. On the less affected side, a notable decrease in iEMG was observed in the ReFe muscle, which is unexpected as this muscle typically benefits from tSCS. On the more affected side, both the iEMG and RMS were reduced in the BiFe. These findings indicate that tSCS may not have been sufficiently effective in improving motor output on both sides, possibly due to the lesion’s location (C4 level), which may prevent effective compensation for the affected motor neurons.

Interestingly, during the session without Exo-H3, muscle activation patterns showed further reductions. A decrease in iEMG was observed in the ReFe and TiAn muscles on the less affected side, and RMS values were lower in the less affected ReFe and GlMe muscles. These results could suggest that the use of the Exo-H3 did not impact positively on muscle activation. It seems that the patient may have been relying on the exoskeleton for support, rather than engaging in the locomotor task actively. Despite the changes in muscle activation, the participant reported no significant differences in discomfort or fatigue between the sessions, with only a slight increase in fatigue during the Exo-H3 session. This aligns with previous studies suggesting mild increases in fatigue with the use of assistive devices (30). Additionally, while the TUG performance improved slightly after both sessions, this change was minimal and likely attributable to the training effect rather than a direct result of the tSCS or Exo-H3 intervention.

The findings suggest that the combined application of tSCS and Exo-H3 did not significantly improve muscle activation or walking performance in Participant 2. This could indicate that the patient’s lack of familiarity with the stimulation or the inability of the stimulation to compensate for the spinal injury’s effects limited the efficacy of both interventions. The lack of improvement could also suggest that the most affected muscles, such as the quadriceps, did not respond as expected to the stimulation, possibly due to the patient’s injury level and the specific characteristics of the spinal lesion. Therefore, further familiarization with the Exo-H3, along with potentially adjusting the stimulation parameters, may be necessary for this patient to achieve better outcomes.

In the case of Participant 3, tSCS appeared to elicit a more favorable neuromuscular response compared to the other subjects. When walking without the Exo-H3, an increase in activation was observed in the ReFe and TiAn muscles on the less affected side, as well as in the ReFe muscle on the most affected side. These findings are consistent with those of Participant 1 and support previous literature identifying the ReFe as a muscle particularly responsive to transcutaneous spinal cord stimulation (15). However, the simultaneous decrease in GaLa activity on the most affected side reflects once again the heterogeneous behavior of this population when exposed to spinal stimulation. Despite the neuromuscular response, functional outcomes in this condition remained modest. The slight improvement in gait speed (0.03 m/s) was below the threshold generally considered to be functionally meaningful (31). Interestingly, although stimulation was perceived as moderately uncomfortable (VAS 4.9 cm), the participant reported no fatigue, suggesting good physical tolerance to the intervention.

During the combined condition with the Exo-H3 and tSCS, limitations in data quality emerged, as signal contamination prevented accurate analysis of ReFe and TiAn activity on the less affected side. Again, problems arise when trying to record EMG during the use of robotic devices due to electromagnetic noise and mechanical artifacts. However, on the most affected side, an increase in RMS was detected in ReFe and TiAn, pointing to a potential synergistic effect when stimulation is applied during robotic-assisted walking. The clinical relevance of this observation is further supported by an 8-s improvement in TUG performance immediately following the session. Although the minimal clinically important difference for TUG in individuals with SCI is estimated at 10.8 s (32), achieving an 8-s gain after a single session is nevertheless encouraging and may reflect functional benefits associated with enhanced neuromuscular recruitments. However, this improvement cannot be attributed to the short intervention alone, as studies with greater number of sessions and a larger sample size are undoubtedly needed. Notably, in this condition the participant reported lower discomfort (VAS 3 cm), although stimulation was perceived as more fatiguing (VAS 4.4 cm), which may reflect greater physical involvement during exoskeleton-assisted walking.

In summary, we identified three potential response types among the subjects. Participant 1 benefited from the application of tSCS alone but did not see improvement with the combined application of tSCS and the Exo-H3. Participant 2 did not benefit from either session, while Participant 3 appeared to benefit from both. This variability may be attributed to the considerable heterogeneity observed in SCI subjects, as outcomes largely depend on the injury’s nature and each patient’s clinical status. However, in patients who exhibited a direct effect of tSCS in terms of muscular activity, the ReFe showed the greatest response, which aligns with previous literature stating that the target muscles for this type of stimulation are mainly the knee extensors (15, 23).

Our results indicate that the combined application of both Exo-H3 and tSCS is feasible, though not uniformly effective for all patients. Importantly, in no case did this approach cause significant discomfort or extreme fatigue beyond what is typically associated with the use of the exoskeleton. While the primary aim of this study was to assess the feasibility of the combined application rather than achieve significant functional gains in a single session, the observed EMG changes in some participants suggest a promising avenue for future research. Specifically, longitudinal studies could explore whether early EMG responses during an initial session may serve as predictors for long-term responsiveness to intensive rehabilitation with the combined approach. Such predictive insights could assist in refining patient selection and personalizing treatment strategies. Further studies, involving a larger sample size and more sessions, are needed to establish criteria for selecting eligible patients who may respond to these rehabilitation therapies.

Given this context, the obtained results raise questions, as they are not consistent across patients. Despite adhering to the same protocols for EGT and tSCS application that we have consistently employed for years, in alignment with established literature, we found contradictory findings. Gorgey et al. (33) reported a single case of a patient trained over 24 sessions (12 weeks), where they found temporal and rhythmic enhancements of the quadriceps and hamstrings EMG, as well as a reduction in exoskeleton assistance. Sutor et al. (15) tested 3 participants with chronic SCI over 12 weeks. They observed individualized responses but a general increase in knee extensor (vastus lateralis) activity during therapy. They also found significant variability in distal muscles (gastrocnemius and soleus), leading to inconclusive results. However, these data were obtained at the post-intervention assessment in both cases, therefore with the patients having mastered the use of the exoskeleton and most of them with a complete motor AIS grade, whereas we report an investigation on the immediate effects and with motor incomplete patients (AIS C or D).

Overall, the combination of tSCS and portable exoskeletons has the potential to provide a synergistic rehabilitative effect on restoring walking abilities (12, 34), although individual differences in the state of the spinal locomotor system necessitate personalized strategies for the placement and timing of stimulation. Our study supports these claims. While we have confirmed that the combination of tSCS+Exo-H3 is feasible and without negative effects, we did not observe consistent immediate effects, either during stimulation or immediately after its application. In our case, the use of a wired EMG amplifier may have introduced noise into the channels due to the use of the exoskeleton (electromagnetic noise and movement artifacts).

The variability in responses could be partly explained by the differing walking speeds among patients, which can affect EMG patterns, as suggested by Hortobágyi et al. (35). Moreover, individual differences in clinical status and the ability to adapt to the exoskeleton could not be fully controlled, as is typical in clinical studies involving SCI patients. While the protocol and measurement conditions were consistent for all participants, there is currently no universally accepted metric to define when a patient is fully “familiarized” with the use of an exoskeleton. In our case, participants were not pre-selected based on prior experience with robotic gait training, as the Exo-H3 is not part of the standard rehabilitation program at the hospital. Therefore, the only exposure participants had to the exoskeleton was through this study. As a result, only three out of five individuals were ultimately included in the analysis, since the other two were not able to complete the requested tasks after receiving the same amount of training.

Achieving a synergistic effect requires the user to master the exoskeleton. The difficulties associated with the use of exoskeletons in the initial sessions are well-documented (36), with a highly variable number of sessions needed to master EGT. Inadequate adaptation between the person and the robotic device leads to altered movement patterns (37) and high fatigue (30). Furthermore, a more detailed analysis of the motor response to stimulation using EMG or other techniques for evaluating muscle response during stimulation is necessary. This evaluation can be conducted under more controlled conditions than walking with the exoskeleton (e.g., in a supine position) and/or using more precise muscle activation recording technologies (e.g., wireless EMG, mechanomyography, or ultrasound), along with a protocol for placing smaller electrodes distributed over the dorso-sacral area.

### Limitations and future lines

This study faced limitations that should be considered when interpreting the results and would be addressed in future research. First, we were unable to analyze trunk muscles involved in the control of the torso due to the incompatibility of surface EMG with the exoskeleton in this body area. Future investigations may consider the use of intramuscular EMG to assess these muscles more effectively in this scenario (38). Additionally, the use of wireless surface EMG systems could significantly improve signal quality by reducing motion artifacts and increasing participant comfort during overground gait training. Complementary sensors such as inertial measurement units (IMUs) could also be incorporated to provide additional data on movement performance and postural control, enriching the analysis of functional improvements.

Second, the approach used for gait segmentation may have impacted the results. Despite the algorithm used for gait segmentation demonstrated acceptable performance (F1-score > 0.91 and mean root mean square error < 64 ms), no reference standard was available in our trials for direct comparison. Future research could address this by incorporating foot switches at the heel and toe to capture precise gait events and further improve the reliability of gait cycle detection.

One key limitation of this study is the very small sample size, as it represents a series of single cases. Recruitment was particularly challenging, given that the Exo-H3 is not part of the hospital’s standard rehabilitation program. As previously noted, three out of the five initially selected participants were able to complete the required tasks within the available familiarization period. Moreover, multiple statistical tests were conducted without correction for multiple testing, increasing the risk of false-positive findings. Although the results cannot be generalized, this is acceptable given the exploratory nature of the study, whose primary aim was not to statistically test a hypothesis, but to assess the feasibility and safety of the combined tSCS and overground EGT in a real-world clinical setting. The findings serve as an initial step toward identifying short-term neuromuscular responses that could help guide future patient selection and protocol personalization. For future research, increasing the sample size and including patients with varying injury characteristics will be essential to define clearer selection criteria and validate the potential of this hybrid intervention on a larger scale.

Another aspect to be considered is that, although the order of the sessions was randomized, the small sample size may not be sufficient to eliminate potential systematic biases related to learning effects or fatigue. In this preliminary stage, no additional analyses were conducted based on session order, as the limited number of participants would prevent any meaningful interpretation of such effects. However, we acknowledge this as a potential confounding factor and highlight the need to control for session sequence more robustly in future studies with larger cohorts.

Furthermore, although the exoskeleton cadence was set to be the same for all patients, we cannot control the walking speed during free ambulation of these patients due to their medical conditions. This variability in walking speed may affect the EMG signal and could explain the heterogeneity of results, as it is well-established that walking speed influences the muscle activation patterns. However, we could not control for this factor as we aimed to maintain the most natural gait possible within the limitations imposed by each individual’s condition.

Although the protocol and measurement conditions were consistent for all participants, it is inherently difficult to ensure the same level of familiarization with the robotic device across individuals, especially given the absence of a standardized criterion to determine when a patient is fully accustomed to using an exoskeleton. This variability may have influenced the participants’ performance and the effects observed during the sessions.

Lastly, allowing additional time for patient familiarization with both the exoskeleton and tSCS could be advantageous. Ideally, future protocols should ensure that users are proficient with the robotic exoskeleton and assess neuromuscular responses (i.e., EMG activity patterns) without exoskeleton assistance prior to therapy, as these can provide early insight into individual responsiveness and guide patient selection as well as the personalization of stimulation and training parameters.

Addressing these limitations will help to improve the robustness, the clinical relevance and the applicability of non-invasive spinal stimulation for neural rehabilitation after SCI.

## Conclusion

This study provides additional evidence supporting the technical feasibility and safety of combining tSCS with overground EGT in patients with incomplete spinal cord injury (SCI). Short-term, session-based responses to the combined application were explored in a real-world clinical setting. Observations of immediate neuromuscular effects and functional performance during routine rehabilitation practice have the potential to provide practical guidance for future customization and refinement of this hybrid intervention to better meet individual needs. The results reveal significant inter-individual variability, underscoring the importance of personalized rehabilitation approaches to maximize therapeutic efficacy. Notably, the ReFe muscle exhibited increased activation in participants who showed immediate effects due to tSCS, which aligns with prior research suggesting that knee extensors may be especially responsive to spinal stimulation. However, consistent immediate effects across participants were not observed, likely due to varying injury characteristics and individual subject profiles. The absence of adverse events indicates that the combined application of tSCS and EGT is safe and tolerable. Rather than aiming to statistically confirm a hypothesis, these preliminary results serve to inform and guide future research focused on refining patient selection criteria and optimizing stimulation parameters through repeated interventions and larger, more diverse samples.

## Data Availability

The raw data supporting the conclusions of this article will be made available by the authors, without undue reservation.
